# Quantitative evaluation of striatal uptake ratios using an adaptive template registration method for ^123^I-ioflupane dopamine transporter SPECT

**DOI:** 10.1007/s12149-024-01968-8

**Published:** 2024-08-19

**Authors:** Sunao Mizumura, Naoyuki Tamamura, Junya Ebina, Hikaru Watanabe, Masaaki Hori

**Affiliations:** 1 Department of Radiology, Toho University Omori Medical Center, 1‑1‑5, Omori‑nishi, Ota‑ku, Tokyo, 143‑8541 Japan; 2 Nihon Medi-Physics Co., Ltd., 3‑4‑10, Shinsuna, Koto‑ku, Tokyo, 136‑0075 Japan; 3 Department of Neurology, Toho University Omori Medical Center, 1-1-5 Omori‑nishi, Ota‑ku, Tokyo, Japan

**Keywords:** ^123^I-Ioflupane, Adapt template registration method, Morphological normalization, Striatal uptake ratio

## Abstract

**Introduction:**

^123^I-FP-CIT (^123^I-Ioflupane) SPECT shows strong accumulation in the striatum, but morphological standardization is challenging due to low accumulation outside the striatum, particularly in subjects with marked striatal decline. In this study, morphological standardization without MRI was achieved using the adaptive template registration (ATR) method to create a subject-specific optimized template with weighted images of normal-type and egg-shape-type templates. The accuracy of a quantitative method for calculating the ratio with nonspecific accumulation in the occipital lobe was evaluated by placing voxels-of-interest (VOI) on standardized images, particularly targeting the striatum.

**Methods:**

The average images of eight subjects, demonstrating normal-type and egg-shape-type tracer accumulation in ^123^I-Ioflupane SPECT, were utilized as normal and disease templates, respectively. The study included 300 subjects that underwent both ^123^I-Ioflupane SPECT and MRI for the diagnosis of suspected Parkinson's disease or for exclusion diagnosis. Morphological standardization of SPECT images using structural MRI (MRI-based method) was considered the standard of truth (SOT). Three morphological standardizations without MRI were conducted. The first involved conventional morphological standardization using a normal template (fixed template method), the second employed the ATR method, with a weighted template, and the third used the split-ATR method, processing the left and right striatum separately to address asymmetrical accumulation. VOIs were set on the striatum, caudate, putamen as regions of specific accumulation, and on the occipital lobe as a reference region for nonspecific accumulation.

**Results:**

Results showed significant and robust linearity in the striatal accumulation ratios for all templates when compared with the occipital lobe accumulation ratio when using the MRI-based method. Comparing intra-class correlations for different linearities, the ATR method and split-ATR method demonstrated higher linearity in the striatum, caudate, and putamen. The split-ATR method showed similar improvements, although more linearity than some of the ATR methods; the effectiveness of the Split-ATR method may vary by image quality, and further validation of its effectiveness in diverse asymmetric accumulation cases seemed warranted.

**Conclusion:**

The use of optimized templates, such as the ATR and split-ATR methods, improved reproducibility in fully automated processing and demonstrated superior linearity compared to that of MRI-based method, in the ratio to the occipital lobe. The ATR method, which enables morphological standardization when using SPECT images only, proved highly reproducible for clinical quantitative analysis of striatal accumulation, facilitating its clinical use.

## Introduction

Dopamine transporter (DAT)-single-photon emission computed tomography (SPECT) is an effective imaging diagnostic method for parkinsonian syndromes, such as Parkinson's disease and Lewy body dementia [1, 2]. While the visual approach of ^123^I-Ioflupane SPECT is generally accepted and preferred for DAT scan analyses, quantitative analysis is highly effective for cross-sectional or longitudinal evaluation. In quantitative analysis in positron-emission tomography (PET) studies, the ratio of accumulation in the striatum relative to a reference region showing nonspecific binding is commonly used [3–7].

To measure the accumulation ratios using a region-based approach with guaranteed reproducibility, methods that assess PET images directly or PET images co-registered with magnetic resonance (MR) images and with automated morphological standardization have been proposed. A typical approach involves using statistical parametric mapping (SPM) to standardize PET data spatially to the Montreal Neurological Institute (MNI) atlas space. While these methods require high-resolution MR scans, which is typically available in research settings, with automated quantification tools designed for routine clinical use, it is beneficial to use PET-only methods, which avoids dependence on the availability of MR scans. This is particularly useful for studies of neurodegenerative diseases using cerebral blood-flow (CBF) SPECT and ^18^F-fluoro-deoxyglucose (FDG) PET, where employing a single normal template can accurately reflect brain accumulation patterns in patients as compared to normal subjects.

However, ^123^I-Ioflupane selectively accumulates in the striatum, showing extremely low accumulation in the cerebral cortex. Moreover, the severity and the distribution of extreme accumulation in the brain vary depending on the pathology: from accumulation limited to the head of the caudate nucleus to partial low accumulation in the putamen. This makes morphological standardization challenging, particularly when setting reference regions for nonspecific accumulation, such as the cerebellar cortex or occipital lobe [8–10].

Similar to DAT-SPECT, morphological standardization analysis using a single template is challenging in amyloid PET, where positive and negative subjects show different cortical accumulation of tracer. However, in amyloid PET, an Adaptive Template Registration (ATR) method has been proposed as a solution, where templates for amyloid-positive and -negative subjects are prepared for analysis of ^18^F-flutemetamol PET image data. Several weighted images are created from these templates, and the most appropriate weighted image is selected as the template for standardizing PET images, with successful outcomes [11–15].

In this study, the adaptive atlas method uses a weighted average image of typical positive and negative images as the template image for morphological standardization. In this study, similar to in amyloid PET image processing, we created representative normal images (normal template images) and abnormal images (egg-shape template) for ^123^I-Ioflupane SPECT. We assumed that images in individual subjects could be approximated using these two templates' weighted images and calculated striatal uptake ratios using localized voxels-of-interest (VOI) on the standardized images. This allowed for quantitative analysis solely from SPECT images, without requiring MR images. We verified the accuracy of this method as compared to that of morphological methods using MR imaging (MRI). In addition, we evaluated the validity of the conventionally used Striatal Binding Ratio (SBR) using the Southampton method by comparing it with the striatal accumulation ratio using the ATR method.

## Subjects and methods

### Subjects

A total of 328 subjects underwent ^123^I-Ioflupane SPECT for suspected Parkinson's disease or Lewy body dementia. Within 6 months thereafter, they underwent MRI. Two certified nuclear medicine specialists, recognized by the Japan Society of Nuclear Medicine (SM, WH), visually interpreted the obtained ^123^I-Ioflupane SPECT images and classified the striatal accumulation distribution into five subgroups: normal-type, eagle-wing-type, egg-shape-type, mixed-type, and burst striatum-type, through consensus [16]. A normal template was created using the average images obtained from eight subjects who were determined to be normal after a light observation without PD symptom than among the normal-type accumulations. Since the burst striatum-type, considered to reflect the most severe condition, did not allow the shape of the striatum to be defined clearly, a disease template was created using the averaged image of eight subjects with Parkinson's disease, with egg-shape-type accumulation.

Among the remaining 312 subjects, excluding those with poor SPECT image quality or segmentation defects in cortical and gray matter on MRI, 300 subjects (aged 49–93 years, 171 females, 129 males) were classified into normal-type (136 subjects), eagle-wing-type (42 subjects), egg-shape-type (86 subjects), mixed-type (23 subjects), and burst striatum-type (13 subjects). Clinical diagnoses for these subjects were as follows: The normal-type included 33 subjects with Alzheimer's disease or mild cognitive impairment, 22 subjects with drug-induced parkinsonism, five subjects with vascular parkinsonism, nine subjects with essential tremor, and 67 subjects with autonomic symptoms observed but no parkinsonian syndrome development during a 2-year follow-up. The eagle-wing-type included 24 subjects with Parkinson's disease, 12 subjects with Lewy body dementia, three subjects with corticobasal syndrome, two subjects with multiple system atrophy, and one subject with drug-induced parkinsonism. The egg-shape-type included 70 subjects with Parkinson's disease, seven subjects with Lewy body dementia, one subject with corticobasal syndrome, two subjects with multiple system atrophy, and two unclassified subjects. The mixed-type included 17 subjects with Parkinson's disease, four subjects with Lewy body dementia, one subject with corticobasal syndrome, and one subject with multiple system atrophy. The burst striatum-type included eight subjects with Parkinson's disease and five subjects with Lewy body dementia.

### Imaging

#### SPECT

Subjects were injected with 167 MBq of ^123^I-Ioflupane. Three hours later, they were remained in a quiet supine position for 30 min, after which image data were acquired. Images were collected using a triple-head GCA-9300R SPECT camera (Canon Medical Systems, Tochigi, Japan) equipped with a fan beam high-resolution (FANHR/N2) collimator (full width at half maximum; FWHM 8.5 mm). Ninety projection images were obtained in 360°, involving five rotations with 30 steps for each 120° imaging of the head. Each step took 12 s. The rotation radius was minimized for each subject. The matrix size was 128 × 128, zoom was 1.00, and the pixel size was 1.72 mm. Counts were acquired within a 30% symmetric energy window centered at 159 keV. The data were reconstructed by filtered back projection using a Butterworth filter (order 4, cutoff frequency 0.65 cycles/cm) as the pre-filter. The images were reconstructed using a ramp filter without attenuation and scatter correction.

#### MRI

MR images were obtained using a 1.5-T Toshiba EXCELART™ Vantage scanner (Canon Medical Systems). Initially, a sagittal T1-weighted field echo sequence (TE = 14 ms, TR = 500 ms, field-of-view = 240 mm, slice thickness = 6 mm, gap = 0.6 mm, 15 slices) was acquired to eliminate the presence of atrophy and other lesions in the brain. Subsequently, a T1-weighted fast field echo sequence (TE = 5.5 ms, TR = 24.4 ms, flip angle = 35°, field-of-view = 240 mm, 256 × 256 matrix) was acquired, with slices placed along the anterior–posterior commissure (AC-PC) line.

### Image procession

#### MRI-based and template-based morphological normalization

To validate the accuracy of morphological standardization using the ATR method, three methods were implemented for morphological standardization using only SPECT data, and employing MRI data as the standard of truth (SOT). These methods included the conventional standardization method using a normal template, as well as two ATR methods. The processing steps for each are described below.

##### MRI-based ^123^I-Ioflupane SPECT standardization method

For this MRI-based method, MRI and SPECT co-registration was performed using SPM8 software (https://www.fil.ion.ucl.ac.uk/spm/) [16–19]. Morphological standardization was then conducted using DARTEL on MRI, and the obtained flow field was used to standardize the SPECT images morphologically. Since this transformation was based on morphological information, it was considered the SOT.

##### Morphological standardization using a normal SPECT template

**Fig. 1:**
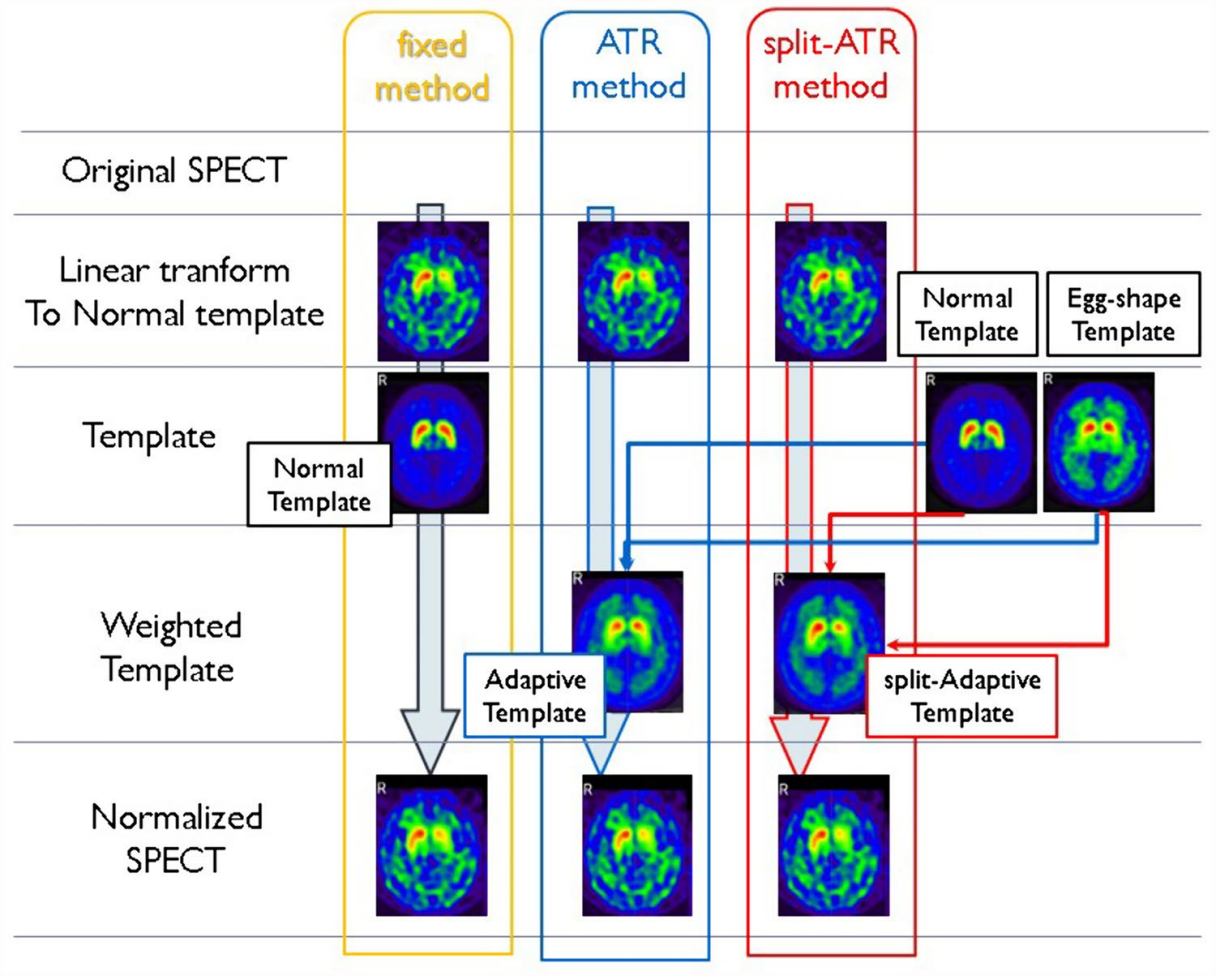
Image Processing Flowchart of Adaptive Template Registration. A flowchart illustrating the image processing steps of the adaptive template registration (ATR) method. Representative normal-type images (8 subjects) and egg-shape-type template images (8 subjects) were standardized to occipital lobe accumulation, and averaged images for normal-type and egg-shape-type templates, respectively, were generated. The fixed method utilized the normal-type template for morphological standardization, in a conventional approach. In the ATR method, weighted images were created using normal-type templates and egg-shape-type templates, respectively, at ratios ranging from 0.1% to 99.9%. The optimization process involved gradually adjusting the weights by 0.1% increments to maximize alignment with the subject's SPECT image. The optimized template was then used for morphological standardization. The split-ATR method processed left and right striatum accumulations separately, using the resulting asymmetrical templates for morphological standardization

For the fixed template method, eight SPECT images classified as normal-type were morphologically normalized by the MRI–SPECT method. Then, pixel values were normalized based on occipital lobe accumulation to produce an average image (creation of the occipital lobe VOI is described later). The obtained average image was used as the normal template. Morphological standardization using SPM8 was then performed using this normal template (Fig. 1). This morphological standardization method using a normal template has been widely used for standardization in CBF-SPECT and FDG-PET studies.

##### Adaptive template registration method

It is important to note that the most severe accumulation-type in ^123^I-Ioflupane SPECT is the burst striatum-type. However, since the margin of the striatum cannot be identified in these images, we used SPECT images with typical abnormal findings, i.e., the egg-shape-type. Similar to the creation of the normal template, we applied morphological standardization using an MRI-based method and accumulation standardization to occipital lobe accumulation on eight SPECT images identified as showing egg-shape-type accumulation, creating an average egg-shape image. Then, after linear transformation of the subject images to the normal template in advance, a weighted average image of the subjects was created, at 0.1% intervals from 99.9% to 0.1% for the normal template and from 0.1% to 99.9% for the oval template. The similarity between the weighted average images and the original SPECT images was evaluated using zero-mean normalized cross-correlation (ZNCC) [20]. The weighting image with the highest similarity was adopted as the optimal template for any given subject for analysis. Morphological standardization was then performed using the adopted individualized template for each target subject (Fig. 1).

##### Split-adapt template registration method

In the aforementioned ATR method, both the normal template and the disease template exhibit almost symmetrical distributions. However, in Parkinson's disease, striatal accumulation is often asymmetrical. Adopting a symmetrical subject template poses a risk of underestimating asymmetry in the striatal accumulation during morphological standardization. Therefore, for the production of subject templates, in order to optimize both left and right striata separately and to improve the accuracy of evaluating asymmetry in striatal accumulation, an asymmetrical subject template was produced and adopted as the template for morphological standardization. The created asymmetrical template was used to perform morphological standardization using the same process as the ATR method (Fig. 1).

### Measurement of striatal accumulation ratio and asymmetry

**Fig. 2:**
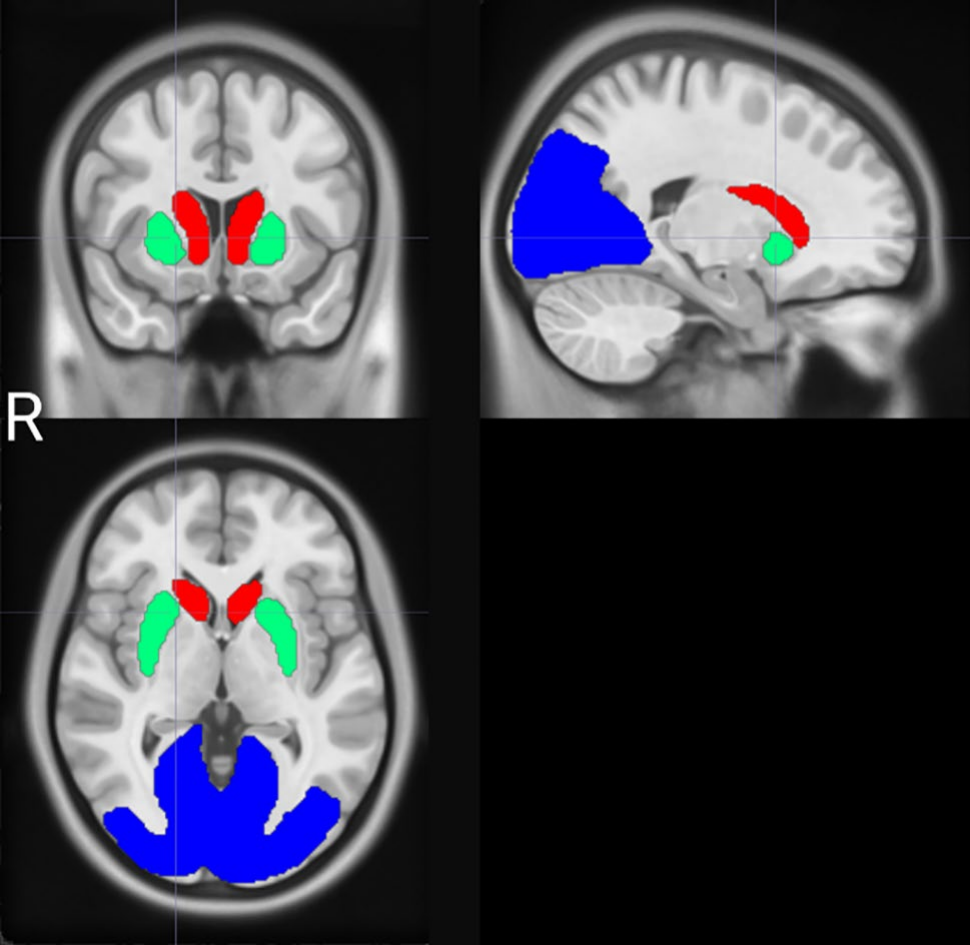
Automated Anatomical Labeling Volumes-of-Interest. Extracted volumes-of-interest (VOI) for the caudate nucleus (Caudate) and putamen (Putamen) were obtained from the "Automated Anatomical Labeling (AAL1) atlas." Regions-of-interest for the occipital lobe were extracted from the regions Occipital_Inf, Occipital_Mid, Occipital_Sup, Lingual, Cuneus, and Calcarine. The combined regions were used as the region-of-interest for the occipital lobe. The VOI was created, displaying the caudate nucleus in red, the putamen in green, and the occipital lobe in blue

For quantitative evaluation of morphological standardized SPECT using the above four methods, VOIs were set at each of the striatum, head of the caudate nucleus, putamen, and the reference region, which was the cerebellum, by MRI-based methods. The stereotactic VOI in the striatum, the caudate nucleus, putamen, and occipital lobe was made using the Automated Anatomical Labeling (AAL1) atlas [21]. The caudate nucleus and putamen were extracted, and the regions of the occipital lobe (Occipital_Inf, Occipital_Mid, Occipital_Sup, Lingual, Cuneus, Calcarine) were extracted for the cerebellum, and were combined to form the VOI for the occipital lobe. If the pixel value of the ROI extracted from the AAL atlas is set to 100 and a 3D-Gaussion filter with a FWHM of 8 mm is applied to match the spatial resolution of SPECT, the value of the VOI edge will decrease. If the pixel value is less than 50, the possibility of belonging to the original VOI can be regarded as less than 50%, so the VOI can be created according to the spatial resolution of SPECT by binarizing only the pixels that are more than 50% excluding these (Fig. 2).

The accumulation ratio for each region was calculated as the ratio of its accumulation to the nonspecific accumulation in the occipital lobe. Accumulation in the striatum, putamen, and head of the caudate nucleus differed between the left and right sides, particularly on the side with less accumulation, the DAT density was lower, indicating more severe impairment. On the side with higher accumulation, normal or milder impairment was presumed. Therefore, the accumulation ratio was separately calculated for the lower and higher accumulation sides. The asymmetry index (AI) was calculated for the left–right difference in accumulation as |the left—the right| / (the left + the right) × 200%. For each of the accumulation ratios of the striatum, the caudate nucleus, and the putamen relative to the occipital lobe and the AI values, accuracy was compared among the MRI-based method and the standardization methods (fixed method, ATR method, and split-ATR method), using correlation analysis for assessment of linearity (Pearson’s regression coefficient) and intra-class correlation for robustness of correlation. The significance level was set at 0.05 for comparison and discussion.

All statistical analyses were performed with EZR (Saitama Medical Center, Jichi Medical University, Saitama, Japan), which is a graphical user interface for R (The R Foundation for Statistical Computing, Vienna, Austria) [22].

### Comparison with the Southampton method

In order to examine the validity of this method, a comparison was made with the previously used Southampton method. For the Southampton method, the striatal accumulation ratio Specific Binding Ratio (SBR) was calculated using DaTView software (Nihon Mediphysics, Co. Ltd., Tokyo, Japan). Since the Southampton method subtracts one from the striatal accumulation ratio to the background accumulation in order to correct for nonspecific accumulation, the striatal accumulation ratio to the occipital lobe obtained by the present method should also be subtracted one for comparison. The correlation analysis was performed as Binding Ratio.

## Results

**Fig. 3:**
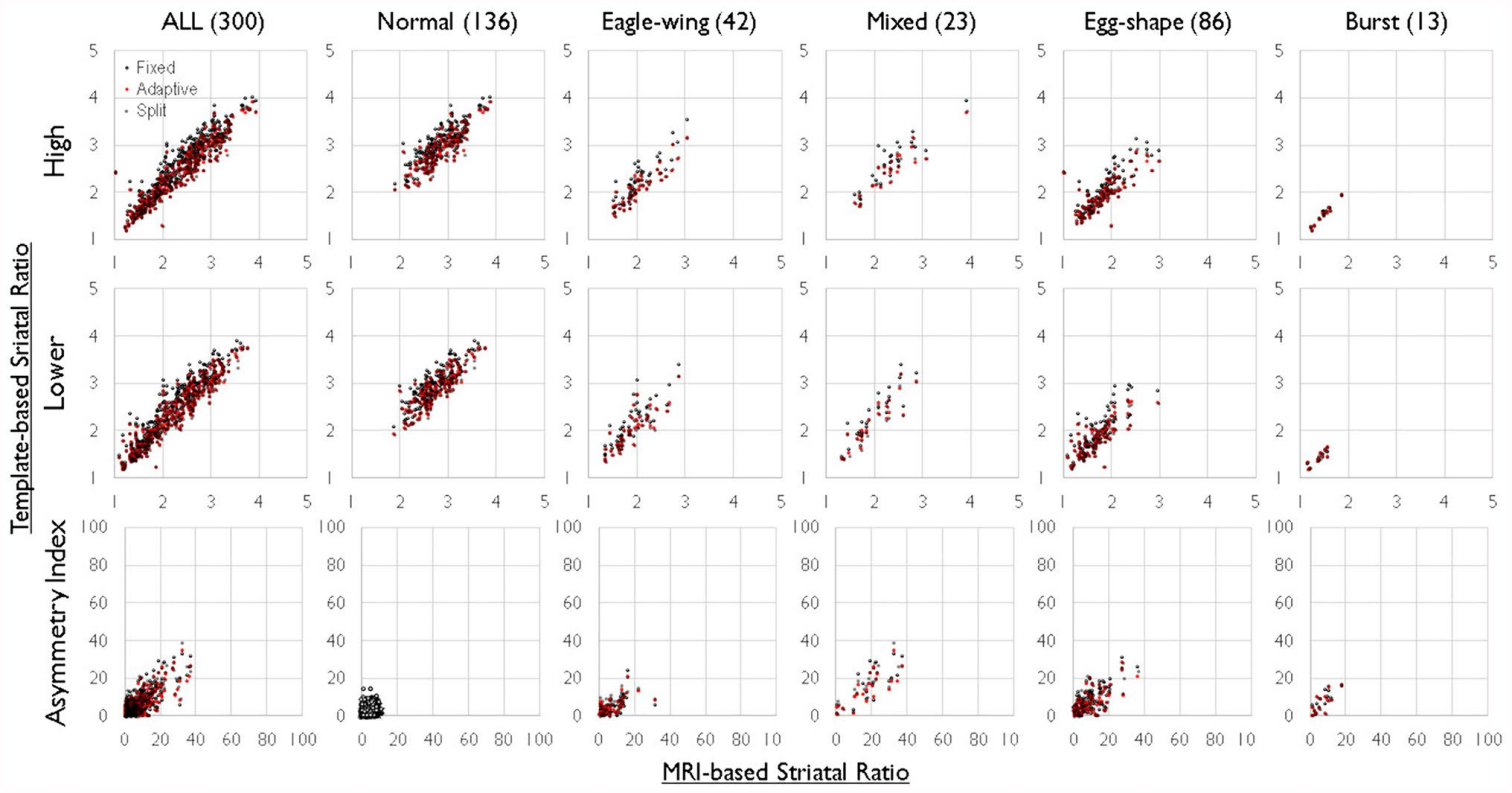
Correlation Analysis of the Occipital Accumulation Ratio and Asymmetry Index (AI) in the Striatum. The upper panel represents scatter plots of the accumulation ratio for high (High) and low (Low) striatal accumulation values in both left and right striata. The X-axis represents the accumulation ratio of the striatum in standardized images using the magnetic resonance imaging (MRI)-based template, while the Y-axis plots three-types of accumulation ratios using the normal template, adaptive template, and split-adaptive template. The lower panel displays a scatter plot of AI values with the X-axis representing the MRI-based template and the Y-axis representing the three-types of accumulation ratios. High striatal accumulation reflects a normal condition or minimal affected status, whereas low striatal accumulation suggests a strongly affected status. Both high and low striatal accumulation ratios exhibit statistically significant linearity. However, in-types with minimal lateral asymmetry, such as normal-type, AI values are small and concentrated near the origin, making the linearity difficult to confirm

**Fig. 4:**
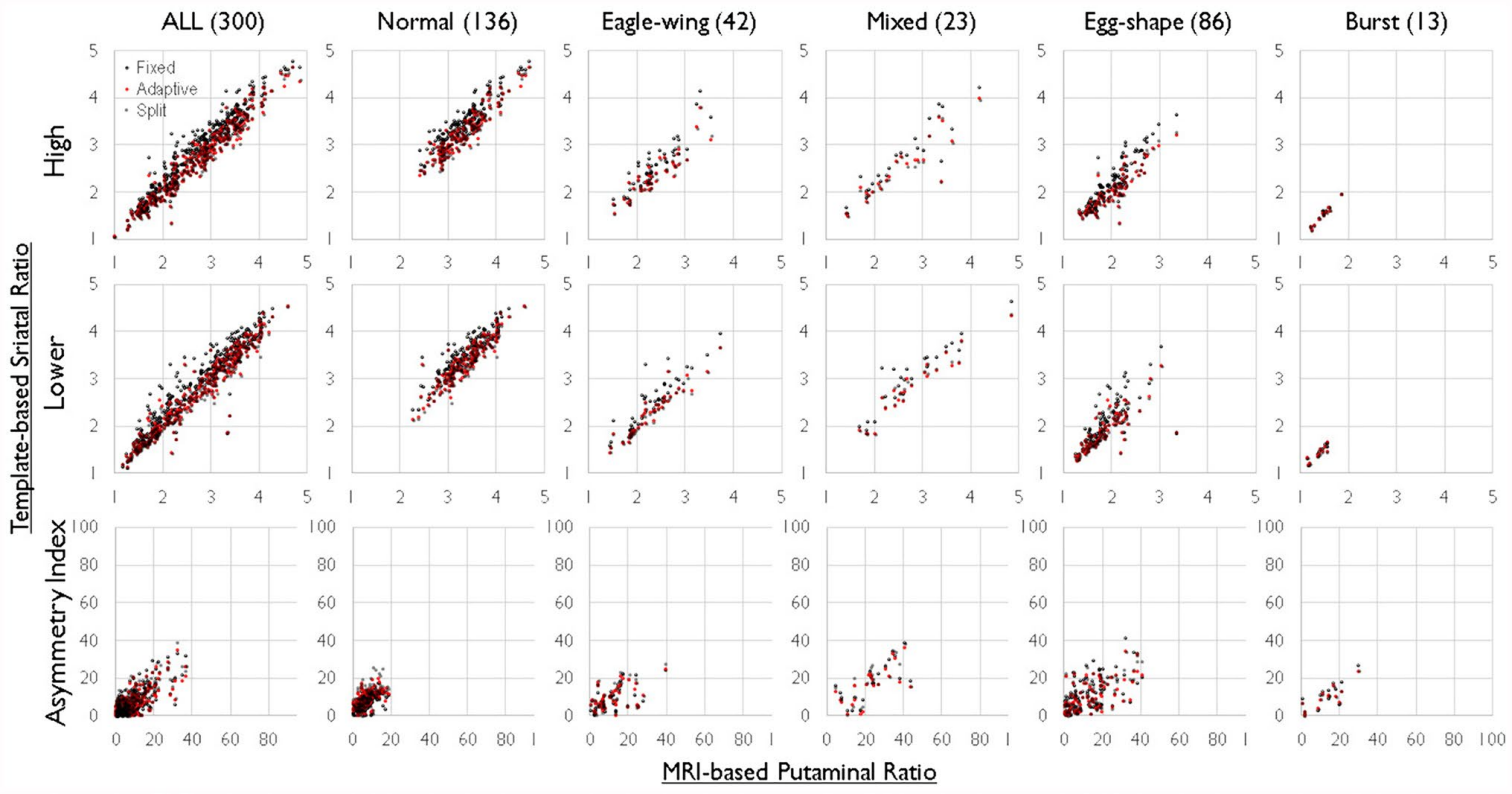
Correlation Analysis of Occipital Accumulation ratio and Asymmetry Index (AI) in the Putamen. The upper panel shows scatter plots of the accumulation ratio for high (High) and low (Low) accumulation values in both the left and right putamen. The X-axis represents the putamen's accumulation ratio in standardized images using the magnetic resonance imaging (MRI)-based template, while the Y-axis plots three types of accumulation ratios using the normal template, adaptive template, and split-adaptive template. The lower panel displays a scatter plot of AI values with the X-axis representing the MRI-based template and the Y-axis representing the three-types of accumulation ratios. The scatter plot of putamen accumulation ratio also exhibits robust linearity, closely resembling the plot of the striatal accumulation ratio (Fig. 3), indicating a significant influence of overall striatal accumulation on putamen accumulation of tracer

For the striatum, caudate, and putamen, scatter plots illustrating the relationship between measurements based on MRI-based methods and measurements based on three different templates were presented for all subjects and each of the five accumulation subgroups (Figs. 3, 4and5). The measured values of linear correlation for each template method are shown in Table 1. Statistically significant linear correlations were confirmed for measurements using any of the templates, demonstrating consistent accuracy across the three measurement methods. To investigate differences in measurement accuracy among the three morphological standardization methods, intra-class correlation coefficients ICC (1, 1) were calculated (Table 2).

**Fig. 5:**
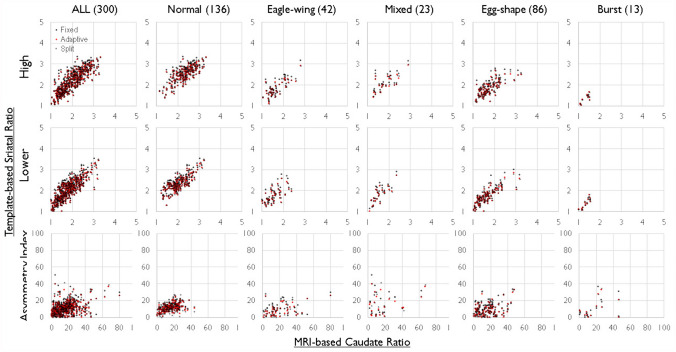
Correlation Analysis of Occipital Accumulation Ratio and Asymmetry Index (AI) in the Caudate. The upper panel shows scatter plots of the tracer accumulation ratio for high (High) and low (Low) caudate accumulation values in both the left and right caudate. The X-axis represents the caudate's accumulation ratio in standardized images using the magnetic resonance imaging (MRI)-based template, while the Y-axis plots three types of accumulation ratios using the normal template, adaptive template, and split-adaptive template. The lower panel displays a scatter plot of AI values with the X-axis representing the MRI-based template and the Y-axis representing the three types of accumulation ratios. The caudate shows a strong linear relationship in accumulation ratios for all-types. However, due to significant lateral asymmetry in caudate accumulation, the AI exhibits linearity as the plot data are widely distributed

**Table 1:** Correlation analysis on of occipital accumulation ratio and asymmetry index (AI)

Correlation analysis vs MRI-based			Striatum				Putamen				Caudate			
			Slope	Intercept	Correlation coefficient	*P* value	Slope	Intercept	Correlation coefficient	*P* value	Slope	Intercept	Correlation coefficient	*P* value
All (*n* = 300)		Fixed	0.972	0.300	0.942	3.56E−143	0.967	0.278	0.966	8.07E−178	0.960	0.290	0.956	3.68E−161
	High	Adaptive	0.926	0.244	0.945	6.40E−147	0.938	0.139	0.971	1.19E−187	0.902	0.293	0.959	1.35E−164
		Split	0.932	0.239	0.945	3.14E−146	0.943	0.135	0.970	3.60E−185	0.900	0.299	0.958	8.07E−164
		Fixed	1.000	0.273	0.940	1.14E−141	0.982	0.228	0.961	4.60E−168	0.929	0.422	0.941	2.06E−142
	Low	Adaptive	0.958	0.223	0.947	1.73E−149	0.950	0.125	0.969	2.47E−182	0.861	0.436	0.938	1.64E−139
		Split	0.952	0.230	0.945	9.59E−147	0.947	0.123	0.968	1.30E−180	0.873	0.417	0.940	2.23E−141
		Fixed	0.705	0.940	0.781	5.46E−63	0.705	0.940	0.681	3.19E−42	0.558	11.782	0.721	4.17E−23
	AI	Adaptive	0.620	1.074	0.764	1.13E−58	0.620	1.074	0.644	1.57E−36	0.623	5.304	0.742	5.44E−25
		Split	0.679	1.706	0.760	1.18E−57	0.679	1.706	0.633	2.56E−39	0.624	5.267	0.741	5.60E−25
Normal (136)		Fixed	0.813	0.767	0.845	2.56E−38	0.895	0.510	0.918	1.23E−55	0.889	0.554	0.955	1.54E−72
	High	Adaptive	0.860	0.459	0.868	1.74E−42	0.958	0.084	0.933	3.04E−61	0.862	0.478	0.956	1.81E−73
		Split	0.875	0.422	0.863	1.70E−41	0.976	0.029	0.928	3.06E−59	0.857	0.493	0.956	3.98E−73
		Fixed	0.843	0.711	0.852	1.45E−39	0.905	0.500	0.913	5.28E−54	0.847	0.706	0.950	1.06E−69
	Low	Adaptive	0.867	0.495	0.871	3.95E−43	0.948	0.162	0.922	5.29E−57	0.803	0.692	0.953	2.82E−71
		Split	0.852	0.526	0.864	8.08E−42	0.944	0.164	0.916	6.51E−55	0.814	0.669	0.954	9.56E−72
		Fixed	0.297	1.399	0.399	1.47E−06	0.548	3.529	0.603	8.36E−15	0.459	17.934	0.721	4.17E−23
	AI	Adaptive	0.129	1.892	0.170	4.77E−02	0.541	5.219	0.591	3.66E−14	0.502	14.379	0.742	5.44E−25
		Split	0.218	2.687	0.188	2.86E−02	0.685	4.696	0.563	1.02E−12	0.503	14.322	0.741	5.60E−25
Eagle (n = 42)		Fixed	1.012	0.207	0.882	1.22E−14	1.024	0.163	0.909	7.79E−17	0.982	0.264	0.902	3.52E−16
	High	Adaptive	0.881	0.294	0.901	4.58E−16	0.850	0.339	0.898	7.09E−16	0.863	0.349	0.923	3.28E−18
		Split	0.895	0.275	0.907	1.43E−16	0.868	0.307	0.908	9.87E−17	0.856	0.364	0.921	6.03E−18
		Fixed	1.020	0.241	0.835	6.44E−12	0.994	0.200	0.904	2.44E−16	0.902	0.451	0.902	3.33E−16
	Low	Adaptive	0.917	0.275	0.854	6.20E−13	0.907	0.190	0.952	2.98E−22	0.762	0.583	0.893	1.95E−15
		Split	0.903	0.296	0.845	1.86E−12	0.902	0.194	0.949	1.22E−21	0.778	0.558	0.898	7.84E−16
		Fixed	0.381	2.473	0.552	1.53E−04	0.378	4.702	0.527	3.41E−04	0.438	18.291	0.712	1.25E−07
	AI	Adaptive	0.395	2.495	0.606	2.13E−05	0.352	5.628	0.511	5.37E−04	0.449	13.515	0.716	9.57E−08
		Split	0.431	3.035	0.589	4.11E−05	0.433	5.282	0.543	2.06E−04	0.450	13.549	0.720	7.85E−08
Mixed (n = 23)		Fixed	0.856	0.639	0.889	1.42E−08	0.832	0.742	0.934	7.23E−11	0.830	0.553	0.928	1.82E−10
	High	Adaptive	0.821	0.527	0.918	6.52E−10	0.815	0.537	0.959	5.45E−13	0.726	0.628	0.905	2.88E−09
		Split	0.838	0.513	0.927	2.11E−10	0.827	0.532	0.962	2.46E−13	0.715	0.663	0.902	4.31E−09
		Fixed	1.167	0.015	0.904	3.37E−09	0.891	0.431	0.930	1.44E−10	0.890	0.523	0.968	4.71E−14
	Low	Adaptive	1.059	0.084	0.914	1.12E−09	0.791	0.487	0.906	2.86E−09	0.793	0.610	0.967	6.45E−14
		Split	1.055	0.074	0.902	4.07E−09	0.794	0.456	0.902	3.99E−09	0.812	0.583	0.968	3.74E−14
		Fixed	0.702	3.908	0.788	8.05E−06	0.652	3.381	0.736	6.35E−05	0.551	12.463	0.841	4.97E−07
	AI	Adaptive	0.638	3.247	0.792	6.78E−06	0.618	2.218	0.707	1.61E−04	0.588	6.235	0.823	1.40E−06
		Split	0.701	4.550	0.813	2.41E−06	0.669	3.413	0.707	1.63E−04	0.592	5.608	0.831	9.01E−07
Egg (n = 86)		Fixed	0.919	0.388	0.841	4.08E−24	1.100	0.018	0.918	1.88E−35	0.828	0.483	0.785	3.86E−19
	High	Adaptive	0.716	0.610	0.784	4.27E−19	0.865	0.266	0.886	7.61E−30	0.654	0.687	0.769	5.69E−18
		Split	0.747	0.564	0.791	1.37E−19	0.897	0.214	0.887	6.74E−30	0.650	0.692	0.770	4.88E−18
		Fixed	0.943	0.370	0.801	2.18E−20	0.974	0.261	0.784	4.77E−19	0.932	0.332	0.875	3.62E−28
	Low	Adaptive	0.732	0.588	0.766	9.02E−18	0.748	0.492	0.786	3.18E−19	0.736	0.535	0.864	1.05E−26
		Split	0.707	0.624	0.756	4.20E−17	0.735	0.509	0.789	1.74E−19	0.754	0.507	0.864	9.91E−27
		Fixed	0.610	2.750	0.686	3.16E−13	0.469	6.014	0.570	9.85E−09	0.580	9.094	0.783	5.14E−19
	AI	Adaptive	0.529	2.561	0.668	2.19E−12	0.438	4.560	0.606	6.33E−10	0.558	2.633	0.801	2.24E−20
		Split	0.591	2.798	0.695	1.20E−13	0.516	4.401	0.658	5.86E−12	0.561	2.574	0.805	1.00E−20
Burst (n = 17)		Fixed	1.180	− 0.209	0.985	1.02E−09	0.982	0.081	0.973	2.43E−08	1.111	− 0.107	0.928	4.78E−06
	High	Adaptive	1.158	− 0.203	0.986	6.11E−10	0.879	0.192	0.970	4.58E−08	0.996	0.058	0.900	2.71E−05
		Split	1.162	− 0.208	0.986	5.69E−10	0.885	0.183	0.970	3.95E−08	0.997	0.056	0.899	2.96E−05
		Fixed	1.127	− 0.128	0.969	5.02E−08	1.148	− 0.202	0.966	8.25E−08	1.014	0.019	0.981	3.29E−09
	Low	Adaptive	1.032	− 0.022	0.933	3.26E−06	1.010	− 0.022	0.969	5.22E−08	0.951	0.064	0.975	1.42E−08
		Split	1.035	− 0.027	0.932	3.50E−06	1.011	− 0.024	0.968	5.85E−08	0.956	0.058	0.975	1.47E−08
		Fixed	0.874	1.792	0.832	4.18E−04	0.698	1.865	0.801	1.01E−03	0.767	6.313	0.847	2.62E−04
	AI	Adaptive	0.792	1.895	0.727	4.89E−03	0.601	1.752	0.839	3.39E−04	0.891	− 0.628	0.834	3.95E−04
		Split	0.816	1.824	0.726	4.94E−03	0.605	1.760	0.834	3.93E−04	0.896	− 0.685	0.832	4.19E−04

The accumulation ratios of each region with the occipital lobe showed significant correlations with the results of measurements using any of the templates. Since AI is a quotient of the left–right accumulation ratio, the error was large. Specifically, the correlation coefficient for AI is close to the product of the left and right correlation coefficients, which seems to be a reasonable result

**Table 2:** Intra-class correlation of occipital accumulation ratio and asymmetry index (AI)

Intercalss Correlation vs MRI-based			Striatum			Putamen			Caudate		
			ICC(1,1)	95% Confidential Interval	F-test p-value	ICC(1,1)	95% Confidential Interval	F-test p-value	ICC(1,1)	95% Confidential Interval	F-test p-value
all (n = 300)		Fixed	0.879	0.850–0.902	1.61E−98	0.938	0.923–0.950	1.61E−140	0.745	0.690–0.791	5.76E−55
	High	Adaptive	0.939	0.924–0.951	2.29E−141	0.970	0.963–0.976	1.27E−186	0.811	0.769–0.847	3.85E−72
		Split	0.937	0.922–0.949	2.47E−139	0.969	0.962–0.976	1.73E−185	0.808	0.764–0.844	4.82E−71
		Fixed	0.857	0.824–0.885	1.04E−88	0.939	0.924–0.951	5.28E−141	0.702	0.640–0.755	1.84E−46
	Low	Adaptive	0.927	0.909–0.941	5.11E−130	0.969	0.961–0.975	1.54E−183	0.776	0.727–0.817	2.62E−62
		Split	0.927	0.909–0.941	7.09E−130	0.967	0.959–0.974	2.05E−181	0.777	0.728–0.818	1.89E−62
		Fixed	0.766	0.715–0.809	1.02E−59	0.661	0.592–0.720	1.63E−39	0.232	0.123–0.336	2.31E−05
	AI	Adaptive	0.723	0.664–0.773	2.46E−50	0.603	0.525–0.670	1.66E−31	0.172	0.061–0.280	1.34E−03
		Split	0.753	0.699–0.798	1.02E−56	0.649	0.579–0.710	8.12E−38	0.172	0.060–0.280	1.35E−03
Normal (136)		Fixed	0.687	0.587–0.766	7.71E−21	0.862	0.812–0.900	2.78E−42	0.504	0.367–0.619	1.57E−10
	High	Adaptive	0.857	0.805–0.896	2.98E−41	0.926	0.898–0.947	1.35E−59	0.655	0.548–0.741	1.57E−18
		Split	0.850	0.796–0.891	5.08E−40	0.922	0.892—0.944	5.05E−58	0.650	0.542–0.738	3.20E−18
		Fixed	0.625	0.511–0.717	1.36E−16	0.835	0.776–0.880	2.14E−37	0.438	0.292–0.564	3.99E−08
	Low	Adaptive	0.818	0.754–0.867	9.97E−35	0.922	0.892–0.943	5.73E−58	0.588	0.467–0.688	1.72E−14
		Split	0.817	0.753–0.866	1.25E−34	0.914	0.882–0.938	1.54E−55	0.589	0.468–0.688	1.62E−14
		Fixed	0.307	0.147–0.451	1.28E−04	0.600	0.481–0.698	3.72E−15	0.268	0.105–0.416	7.68E−04
	AI	Adaptive	0.090	-0.079–0.253	1.48E−01	0.507	0.370–0.621	1.22E−10	0.238	0.073–0.390	2.50E−03
		Split	0.019	0.022–0.345	1.34E−02	0.456	0.313–0.580	9.64E−09	0.220	0.054–0.373	4.88E−03
Eagle (n = 42)		Fixed	0.735	0.559–0.848	7.29E−09	0.816	0.684–0.896	8.58E−12	0.594	0.359–0.759	1.13E−05
	High	Adaptive	0.894	0.812–0.941	1.85E−16	0.899	0.820–0.944	7.15E−17	0.715	0.530–0.836	2.66E−08
		Split	0.896	0.816–0.943	1.17E−16	0.909	0.839–0.950	7.53E−18	0.711	0.524–0.833	3.40E−08
		Fixed	0.630	0.408–0.782	2.49E−06	0.846	0.733–0.914	2.61E−13	0.485	0.218–0.685	4.48E−04
	Low	Adaptive	0.894	0.770–0.953	9.64E−10	0.951	0.912–0.974	2.41E−23	0.605	0.373–0.765	7.40E−06
		Split	0.892	0.765–0.952	1.23E−09	0.947	0.904–0.971	1.45E−22	0.600	0.367–0.762	8.96E−06
		Fixed	0.460	0.187–0.668	8.73E−04	0.465	0.193–0.671	7.66E−04	0.046	-0.256–0.341	3.83E−01
	AI	Adaptive	0.501	0.238–0.696	2.81E−04	0.461	0.189–0.669	8.43E−04	0.065	-0.238–0.358	3.38E−01
		Split	0.546	0.295–0.727	6.71E−05	0.527	0.271–0.714	1.27E−04	0.076	-0.227–0.368	3.12E−01
Mixed (n = 23)		Fixed	0.742	0.488–0.881	1.19E−05	0.864	0.710–0.940	1.49E−08	0.415	0.020–0.700	2.03E−02
	High	Adaptive	0.894	0.770–0.953	9.64E−10	0.949	0.884–0.978	3.24E−13	0.575	0.229–0.794	1.40E−03
		Split	0.892	0.765–0.952	1.23E−09	0.951	0.890–0.979	1.82E−13	0.558	0.206–0.784	1.97E−03
		Fixed	0.678	0.383–0.849	1.07E−04	0.911	0.804–0.961	1.45E−10	0.496	0.122–0.749	6.11E−03
	Low	Adaptive	0.822	0.630–0.920	2.55E−07	0.900	0.781–0.956	5.42E−10	0.599	0.263–0.807	8.35E−04
		Split	0.825	0.636–0.922	2.11E−07	0.895	0.772–0.954	8.77E−10	0.608	0.276–0.811	6.77E−04
		Fixed	0.783	0.558–0.901	2.08E−06	0.661	0.357–0.840	1.72E−04	0.301	− 0.111–0.627	7.32E−02
	AI	Adaptive	0.735	0.476–0.878	1.54E−05	0.559	0.207–0.785	1.94E−03	0.282	− 0.131–0.615	8.72E−02
		Split	0.809	0.606–0.914	5.42E−07	0.656	0.348–0.837	2.02E−04	0.247	− 0.168–0.590	1.19E−01
Egg (n = 86)		Fixed	0.686	0.556–0.784	8.61E−14	0.797	0.705–0.863	8.30E−21	0.719	0.599–0.807	1.70E−15
	High	Adaptive	0.756	0.649–0.834	9.29E−18	0.887	0.832–0.925	6.66E−31	0.726	0.608–0.812	6.84E−16
		Split	0.759	0.653–0.836	5.67E−18	0.888	0.833–0.925	5.53E−31	0.726	0.609–0.812	6.72E−16
		Fixed	0.589	0.433–0.712	8.14E−10	0.674	0.540–0.774	3.41E−13	0.764	0.659–0.839	2.79E−18
	Low	Adaptive	0.699	0.572–0.792	2.08E−14	0.786	0.690–0.855	6.78E−20	0.788	0.692–0.856	4.84E−20
		Split	0.692	0.563–0.787	4.69E−14	0.789	0.694–0.857	3.88E−20	0.786	0.689–0.855	6.99E−20
		Fixed	0.680	0.548–0.779	1.79E−13	0.560	0.397–0.689	7.45E−09	0.254	0.047–0.441	8.49E−03
	AI	Adaptive	0.628	0.481–0.740	3.15E−11	0.544	0.378–0.677	2.20E−08	0.122	-0.090–0.324	1.29E−01
		Split	0.682	0.551–0.780	1.38E−13	0.626	0.478–0.739	3.83E−11	0.135	-0.077–0.336	1.06E−01
Burst (n = 17)		Fixed	0.958	0.873–0.987	1.18E−09	0.959	0.876–0.987	9.81E−09	0.905	0.729–0.970	1.93E−06
	High	Adaptive	0.973	0.918–0.992	6.10E−10	0.967	0.901–0.990	2.28E−09	0.886	0.680–0.963	6.21E−06
		Split	0.973	0.917–0.992	6.85E−10	0.969	0.904–0.990	1.80E−09	0.885	0.678–0.963	6.46E−06
		Fixed	0.948	0.846–0.984	4.28E−08	0.959	0.859–0.985	2.35E−08	0.912	0.747–0.972	1.22E−06
	Low	Adaptive	0.930	0.795–0.978	2.92E−07	0.970	0.908–0.991	1.37E−09	0.874	0.651–0.959	1.13E−05
		Split	0.929	0.792–0.978	3.19E−07	0.969	0.906–0.990	1.60E−09	0.874	0.649–0.959	1.17E−05
		Fixed	0.826	0.539–0.943	8.13E−05	0.778	0.436–0.926	3.47E−04	0.439	− 0.103–0.785	5.28E−02
	AI	Adaptive	0.738	0.357–0.911	9.07E−04	0.717	0.316–0.903	1.43E−03	0.270	− 0.289–0.698	1.68E−01
		Split	0.734	0.349–0.910	9.92E−04	0.719	0.321–0.904	1.36E−03	0.274	− 0.285–0.701	1.64E−01

This table presents the results of investigating the correlation between the cerebellar accumulation ratios with high and low tracer accumulation and the asymmetry index (AI) for each distribution-type of striatal accumulation. The accumulation ratios using the ATR and split-ATR methods showed higher ICC (1,1) values than those obtained using the fixed template (highlighted in light blue cells)

ICC (1,1) indicates the robustness of the correlation, showed that the ATR and split-ATR methods improved the robustness in most, but not all, regions of interest and accumulation patterns. However, the ATR method and split-ATR method showed similar ICC (1,1) values, and limited effects in measurement accuracy was found between symmetric and asymmetric template use. Fix: fixed method, Adapt: adaptive template registration method, Split: split-ATR method, High, Low; the accumulation ratio for high and low striatal accumulation values in both the left and right striata, respectively

The accumulation ratios of each region with the occipital lobe showed significant correlations with the results of measurements using any of the templates (Table 1). However, since the superiority or inferiority of the correlation cannot be examined in the correlation analysis, comparison of ICC (1, 1) (Table 2), which indicates the robustness of the correlation, showed that the ATR and split-ATR methods improved the robustness in most, but not all, regions of interest and accumulation patterns. The ATR method and split-ATR method showed similar ICC (1, 1) values, and limited effects in measurement accuracy was found between symmetric and asymmetric template usage (Table 2). Since AI is a quotient of the left–right accumulation ratio, the error was large and ICC (1, 1) was also low. Specifically, the correlation coefficient for AI is close to the product of the left and right correlation coefficients, which seems to be a reasonable result.

**Fig. 6:**
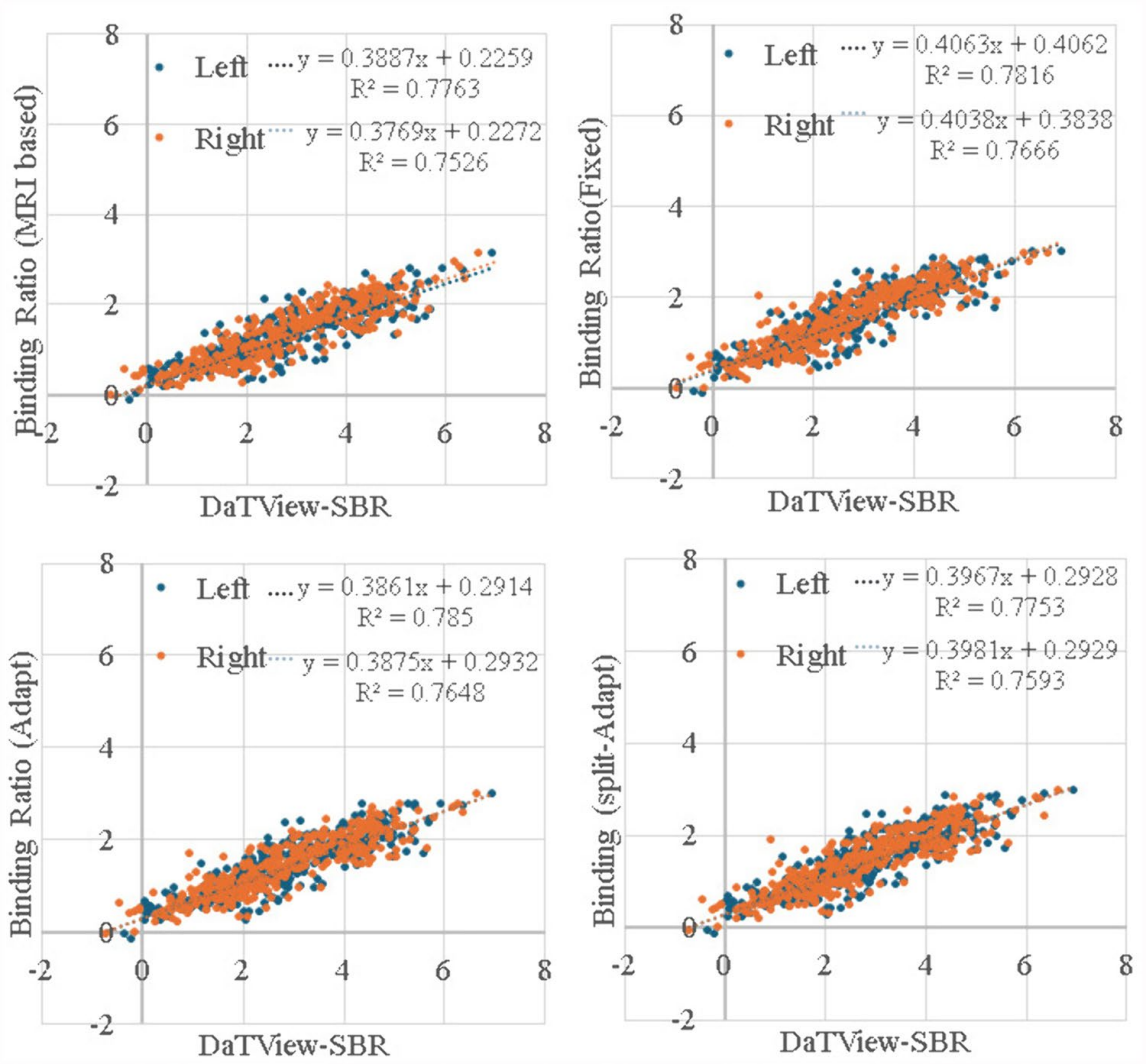
Correlation analysis of striatal uptake ratio between DaTView-Southampton and four Template Methods. Binding Ratio = (Accumulation Ratio to Occipital lobe)—1. Significant linearity was confirmed in all subjects, indicating that this method is also an effective quantitative analysis. However, the SBR value obtained by the Southampton method sometimes takes a negative value, which may include a measurement error, while the ATR method does not have a negative value. Here, the AI is omitted from the display because it does not show a consistent distribution of accumulation without statistical dominance

Comparison of the quantitative values with the Southampton method is shown in Fig. 6 and Table 3. Significant linearity was confirmed in all subjects, indicating that this method is also an effective quantitative analysis. However, the SBR value obtained by the Southampton method sometimes takes a negative value, which may include a measurement error, while the ATR method does not have a negative value.

**Table 3:** Correlation analysis of (**a**) striatal uptake ratio, (**b**) Asymmetry Index between DaTView-Southampton Method and four Template Methods

(a) Occipital lobe uptake Ratio					
Binding Ratio vs DaTView-SBR (n = 323)		Correlation Analysis			
		Slope	Intercept	Correlation coefficient	p- value
MRI-based	Left	0.377	0.227	0.868	2.27.E−99
	Right	0.389	0.226	0.881	2.05.E−106
Fixed Template	Left	0.404	0.384	0.876	1.95.E−103
	Right	0.406	0.406	0.884	4.62.E−108
Adaptive Template	Left	0.388	0.293	0.875	6.68.E−103
	Right	0.386	0.291	0.886	3.67.E−109
split-Adaptive Template	Left	0.398	0.293	0.871	2.75.E−101
	Right	0.397	0.293	0.881	4.30.E−106

(b) Assynmetry Index				
Assymmery Index (n = 323)	Correlation analysis			
	Slope	Intercept	Correlation coefficient	p-value
MRI-based	− 0.003	14.565	0.049	n.s
Fixed Template	− 0.003	10.435	0.047	n.s
Adaptive Template	− 0.004	10.517	0.073	n.s
split-Adaptive Template	− 0.004	12.369	0.073	n.s

Binding Ratio = (Accumulation Ratio to Occipital lobe)—1

*n.s.* no significance

DaTView-SBR = specific binding ratio using DaTView software:

Significant linearity was confirmed in all subjects, indicating that this method is also an effective quantitative analysis. However, the DaTView-

SBR value obtained by the Southampton method sometimes takes a negative value, which may include a measurement error, while the ATR method does not have a negative value

**Fig. 7:**
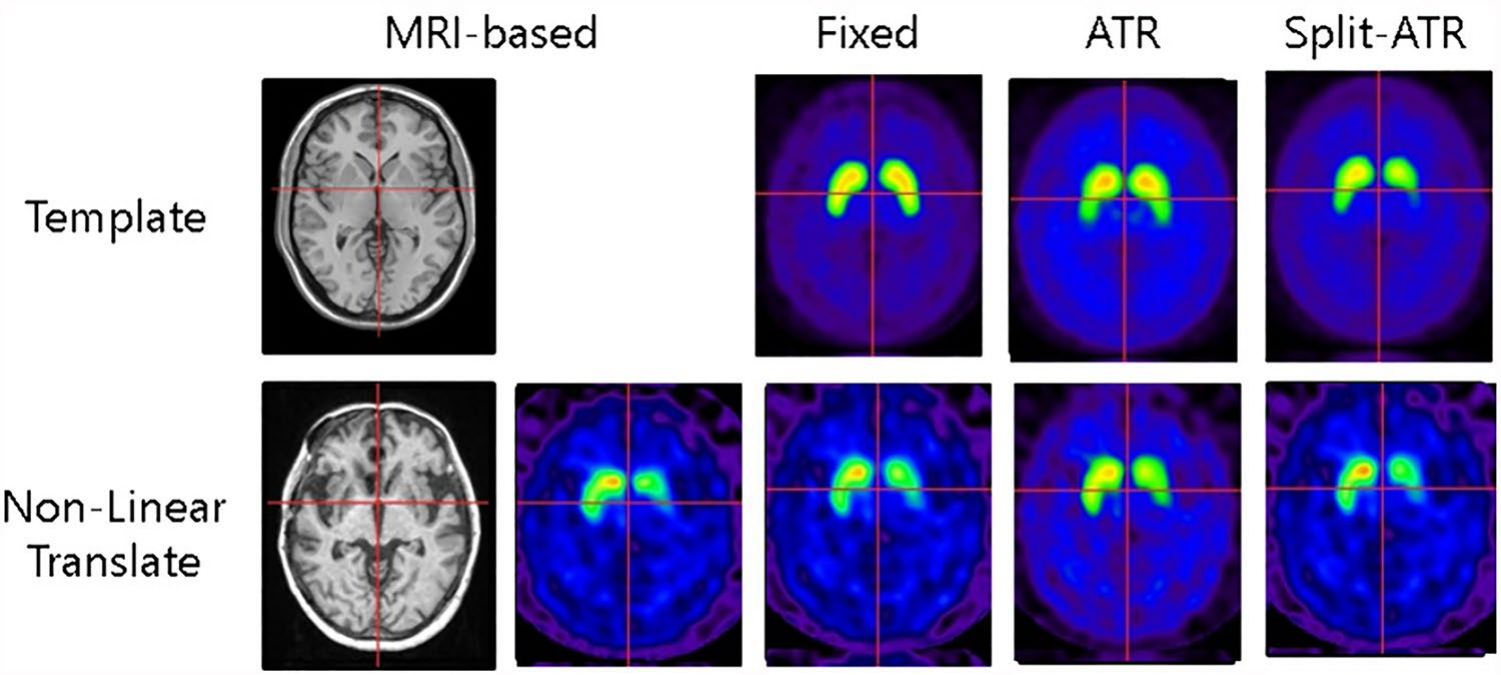
An illustrative example of a case in which the use of an asymmetric template (split-ATR method) proved an efficacious approach to transformation. The DAT image was transformed to align with the standard striatal accumulation (fixed method), yet the left striatum exhibited a posterior shortening, resulting in a posteriorly extended distribution of the left striatum following the transformation. The ATR method, which is based on a symmetric template with a shortened posterior putamen, also produces an underestimation of the right striatal volume and an overestimation of the left striatal volume. This results in a longer posterior left putamen and higher accumulation in the right putamen after conversion. However, the split-ATR method, which uses an asymmetric template, successfully approximates the MRI-Base method even for the low-accumulated left striatum

Figure 7 illustrates the results of image processing in a case exhibiting pronounced asymmetry in striatal accumulation. The three-detector gamma camera image is directly standardized to the normal template, and the striatal distortion is not significant due to the high image resolution. However, the left striatal accumulation is observed to have a shorter posterior putamen than the normal striatum, which results in an oppositely longer capsule after transformation. Even after transforming the image of this case using a symmetric template with a short posterior capsule by the ATR method, the left striatum does not perfectly match the MRI-based image, and the putaminal accumulation is increased because the right putaminal volume is estimated to be smaller. Conversely, the split-ATR method conversion approximates the distribution of the left striatum to the MRI-based image using an asymmetric template.

## Discussion

Two principal methodologies exist for the quantification of DAT scans. One such method is the simultaneous registration of MRI and SPECT data, which facilitates accurate alignment and measurement of SPECT accumulation within the VOI delineated on the MRI image. The alternative approach is the Southampton method, which does not employ simultaneous registration with MRI. In contrast, a comprehensive VOI encompassing the striatum is initially delineated, the accumulation is subsequently quantified, and the striatal accumulation is then calculated by subtracting the nonspecific accumulation. Another method that does not involve simultaneous registration with MRI, DaTQUANT (GE Healthcare, Little Chalfont, UK), has been demonstrated to have excellent quantitative performance [23]. However, specific quantification methods using DaTQUANT have yet to be published.

In Japan, the Southampton method has been widely employed as a quantitative method for ^123^I-Ioflupane SPECT [24, 25]. This method utilizes a pentagon-shaped region-of-interest (ROI) to measure striatal accumulation and nonspecific accumulation around the striatum. The advantage of this method lies in its ability to extract striatal accumulation even from low-resolution SPECT images. However, challenges in measurement accuracy exist, such as incomplete reproducibility due to the manual setting of the pentagon-shaped ROI and the influence of cerebrospinal fluid on nonspecific accumulation in subjects with atrophy. Approaches such as correcting for measurement errors due to atrophy and cerebrospinal fluid have also been presented [26, 27].

In the proposed ATR method, even in subjects with widespread reduction in striatal accumulation, morphological standardization is achievable. The use of morphological standardization enables precise morphological localization, facilitating complete automation of image processing and improved reproducibility. Furthermore, with ATR, not only can the overall striatal accumulation be evaluated, but partial accumulations of the striatum, such as in the head of the caudate nucleus and putamen, can also be assessed. ATR can be easily applied to routine clinical practice as it analyzes SPECT images without the need for MR images. Additionally, as morphological standardization encompasses not only striatal accumulation but also whole brain accumulation, localized VOI using proper image transformation may allow quantitative evaluation of areas outside the striatum, such as the infratentorial region.

### ATR and split-ATR method evaluation

The optimal template used in the ATR method is created from weighted images of the common normal and oval templates and exhibits a symmetric distribution. As a result, symmetric templates may not accurately reflect the asymmetry of striatal accumulation seen in many Parkinson's disease patients. To address this issue, we created optimal templates for the left and right striatum separately and evaluated the effectiveness of the Split-ATR method using asymmetric templates. The results showed that the split-ATR method was more effective in some cases (Fig. 7), and the correction effects obtained with the ATR and split-ATR methods were very similar (Table 2). This means that even if the striatum is visually under-accumulated, the shape of the striatal accumulation is recognized on the image processing.

However, the accuracy of morphological standardization is affected by differences in image quality, such as uniformity and resolution caused by SPECT equipment, data acquisition, and image reconstruction, so the correction effect of the split-ATR method may be different, and the effect of SPECT image quality may also need to be compared. The split-adaptive template method is in principle a simple process that provides quantitative improvement, and a new study design for cases with strong left–right differences may be needed to evaluate its correction effect.

Compared to the accumulation ratio of the striatum, putamen, and head of the caudate nucleus calculated using the MRI-based method, the accumulation ratios obtained with the ATR method and split-ATR method exhibited extremely high linearity. This suggests that, in clinical use, these methods are suitable for a quantitative approach. Reproducibility improvements due to fully automatic processing of quantitative evaluations, and advantages in terms of local evaluation through stereotactic analysis are anticipated.

Although the Southampton method, which is widely used in clinical practice, has different analysis methods and reference regions for non-specific accumulation and thus different values for striatal accumulation ratio, the ATR and split-ATR methods showed a very good and significant correlation with the striatal accumulation ratio by the Southampton method (Fig. 6, Table 3).

The relationship between age and sex for the striatal accumulation ratio to occipital lobe accumulation when using the ATR method in subjects with normal-type accumulation in Appendix. However, the normal-type subjects in this study were not healthy control subjects, and thus, further investigations of healthy control subjects are deemed necessary. Nevertheless, similar to previous reports [28, 29], this method may reflect a gradual decrease in striatal accumulation due to aging.

A limitation of the ATR method is that the initial anatomical standardization of DAT images may be incomplete due to linear transformations, and errors in optimal template selection may still remain when optimal templates are selected from these linearly transformed images. To improve the accuracy of the standardization, the optimal template selection and nonlinearity standardization can be repeated. Considering the resolution of SPECT images and the complexity of image processing, we chose the latter of the two options: improve accuracy by iterative computation and avoid the risk of image distortion due to divergence.

The remaining issue is that morphological standardization may fail in subjects with extreme ventricular enlargement or imaging loss in the lower part of the cerebellum. In the latter case, addressing morphological standardization by excluding the lower part of the cerebellum from the standard brain coordinate range might be a possible solution. However, for morphological peculiarities, such as ventricular enlargement, the method may still have limitations in the adaptability range, similar to statistical analyses of conventional images. Additionally, as mentioned earlier, morphological standardization involves not only the striatum, but also the entire head, including scalp and cranial accumulations. Therefore, SPECT images should be evaluated for the distribution of accumulations in the entire head, without masking out regions other than the striatum, to ensure a comprehensive image assessment.

Furthermore, the size of the striatum shows individual differences, and morphological standardization may result in an inaccurate evaluation of striatal accumulation. For a precise assessment, morphological standardization through MRI is necessary. However, in the clinical context, these variations are unlikely to introduce significant errors and are considered acceptable for clinical use.

## Conclusion

The ATR method enabled morphological standardization without using MR images, by processing weighted images of normal and disease templates. The ease of morphological standardization improved the reproducibility of quantitative, stereotactic evaluations through VOI assessments, making clinical applications straightforward. Even in images with visual asymmetry in striatal accumulation, evaluating the striatal accumulation ratio was possible for most clinical subjects. The image correction effect of the Split-ATR method may vary depending on the quality of SPECT images, and it was inferred that a study specific to cases with left–right differences in striatal accumulation is necessary for its effectiveness.

## Figures and Tables

**Fig. 8:**
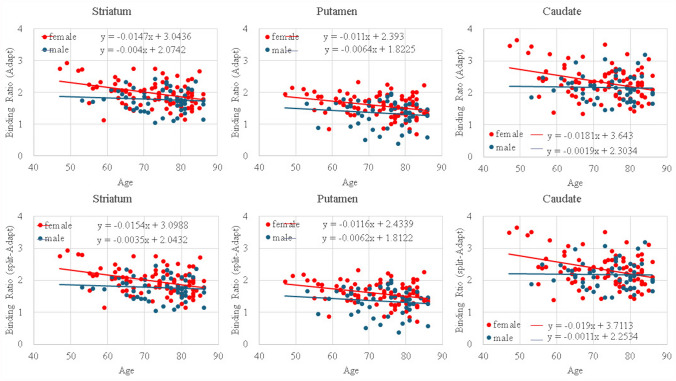
Correlation Analysis between Age and Binding Ratio of Normal-Type Subjects in Adaptive Template and split-Adaptive Template Methods. Binding Ratio = (Accumulation Ratio to Occipital lobe)—1. Scatter plots displaying the Binding Ratio in the striatum, putamen, and caudate for normal-type subjects, with age separated according to sex. All Binding Ration decrease with age, with a faster decline observed in females

## Data Availability

All data used to support the findings of this study are included within the article.

## Appendix

In this study, we present the results of a comparison of the association between the accumulation ratios in the striatum, caudate nucleus and putamen of DAT-SPECT showing normal accumulation distribution by our proposed ATR and split-ATR methods and their association with age and sex (Fig. 8, Tables

**Table 4:** Correlation Analysis between Binding Ratio in the Striatum, Putamen, and Caudate of Normal-Type Subjects and Age

	Sex	Site	Slope	Intercept	Correlation coefficient	*p*-value
Binding Ratio (Adapt)	Female (*n* = 85)	Striatum	− 0.01	3.04	0.36	6.30E−04
		Putamen	− 0.01	2.39	0.31	3.63E−03
		Caudate	− 0.02	3.64	0.34	1.30E−03
	Male (*n* = 51)	Striatum	− 4.03E−03	2.07	0.09	n,s
		Putamen	− 0.01	1.82	0.13	n,s
		Caudate	− 1.89E−03	2.30	0.04	n,s
Binding Ratio (split-Adapt)	Female (85)	Striatum	− 0.02	3.10	0.37	4.71E−04
		Putamen	− 0.01	2.43	0.32	2.61E−03
		Caudate	− 0.02	3.71	0.35	1.01E−03
	Male (*n* = 51)	Striatum	− 3.52E−03	2.04	0.08	n,s
		Putamen	− 0.01	1.81	0.12	n,s
		Caudate	− 1.10E−03	2.25	0.02	n,s

However, AI is omitted from the display because it does not show a consistent distribution of accumulation without statistical dominance

n.s. = no significance Binding Ratio = (Accumulation Ratio to Occipital lobe)—1

A gradual decrease in the rate of striatal accumulation with increasing age was observed (although statistical significance was not reached in males). Moreover, aging had a stronger effect on the head of the caudate nucleus than on the putamen

4 and

**Table 5:** Binding Ratio of Normal-Type Subjects in Adaptive Template and split-Adaptive Template Methods by Age and Sex

Binding Ratio (Adapt)			Striatum		Caudate		Putamen	
	Age	*N*	Ave	SD	Ave	SD	Ave	SD
Female	≤ 69	30	2.10	0.43	1.71	0.30	2.46	0.60
	70–79	30	1.98	0.34	1.56	0.33	2.36	0.43
	80 ≥	25	1.84	0.36	1.50	0.36	2.14	0.42
Male	≤ 69	14	1.81	0.27	1.37	0.37	2.22	0.32
	70–79	21	1.77	0.41	1.38	0.46	2.13	0.41
	80 ≥	16	1.75	0.30	1.30	0.28	2.16	0.45

Binding Ratio (split-Adapt)			Striatum		Caudate		Putamen	
	Age	*N*	Ave	SD	Ave	SD	Ave	SD
Female	≤ 69	30	2.12	0.43	1.72	0.31	2.48	0.61
	70–79	30	1.98	0.35	1.56	0.33	2.36	0.45
	80 ≥	25	1.84	0.37	1.50	0.37	2.14	0.44
Male	≤ 69	14	1.81	0.28	1.37	0.37	2.22	0.33
	70–79	21	1.78	0.42	1.38	0.47	2.14	0.42
	80 ≥	16	1.76	0.31	1.31	0.29	2.18	0.45

Binding Ratio = (Accumulation Ratio to Occipital lobe)—1

This table provides examples illustrating the distribution of tracer accumulation in the striatum in normal-types, listing the mean and unbiased standard deviation of the Binding Ratio for the striatum, putamen, and caudate, according to age and sex. The Binding Ratio decreases with age, and females exhibit a faster rate of decline for all Binding Ration

5). Striatal accumulation ratios were found to decrease with age, as previously reported, reflecting the effects of aging [28, 29]. Furthermore, aging had a stronger effect on the caudate nucleus head than on the putamen.
